# Suicidal behaviours among adolescents in Liberia

**DOI:** 10.1186/s12888-020-02985-3

**Published:** 2020-12-01

**Authors:** Emmanuel Nii-Boye Quarshie, Henry K. Onyeaka, Kwaku Oppong Asante

**Affiliations:** 1 Department of Psychology, University of Ghana, P.O. Box LG 84, Accra, Ghana; 2 School of Psychology, University of Leeds, Leeds, UK; 3 Department of Psychiatry, Massachusetts General Hospital/Mclean Hospital, Boston, USA; 4 Harvard Medical School, Boston, USA; 5 Department of Psychology, University of the Free State, Bloemfontein, South Africa

**Keywords:** Adolescents, Attempted suicide, Liberia, Suicide, Suicidal ideation, Suicidal planning

## Abstract

**Background:**

Whereas suicide remains in the top 12 leading causes of death among young people aged 10–24 in sub-Saharan Africa, little is known about suicidal behaviours among adolescents in Liberia. We aimed to estimate the 12-month prevalence and describe some of the correlates of suicide behaviours (ideation, planning, and attempt) among school-going adolescents in Liberia.

**Methods:**

We analysed data from the 2017 Liberia Global School-based Student Health Survey conducted nationwide among secondary school students. We performed bivariate and multivariable analyses to assess the correlates of suicidal ideation, planning, and attempt in the previous 12 months.

**Results:**

Of the 2744 students, 26.8% reported suicidal ideation, 36.5% made a specific plan to attempt suicide and 33.7% attempted suicide during the 12 months preceding the survey. In the final adjusted logistic models, bullying victimisation and food insecurity were associated with increased odds of ideation, planning, and attempt. Whereas no factor was uniquely associated with suicidal ideation, having many close friends, and parental monitoring were associated with the increased odds of suicidal planning only. Leisure-time sedentary behaviour was associated with increased odds of suicidal planning and attempt. Cannabis use, alcohol drunkenness, being physically attacked, and parental supervision were uniquely associated with increased odds of suicidal attempt, while parental understanding and having a smaller number of close friends were uniquely associated with reduced odds of suicidal attempt.

**Conclusions:**

The relatively high prevalence estimates of suicide behaviours and the multi-contextual nature of the associated factors warrant the need for the design and implementation of universal and multi-level, collaborative targeted intervention efforts towards the prevention of the onset of ideation, planning, and attempt, and the possible transition to deaths by suicide among school-going adolescents in Liberia.

**Supplementary Information:**

The online version contains supplementary material available at 10.1186/s12888-020-02985-3.

## Background

According to the World Health Organization (WHO), suicidal behaviour is “a range of behaviours that include thinking about suicide (or ideation), planning for suicide, attempting suicide and suicide itself” [1]. Globally, suicidal behaviours represent a leading cause of injury and death, with 79% of suicide-related mortality reported in low- and middle-income countries, LAMICs [1–3]. Across the world, suicide represents the second leading cause of death among 15–19-year-olds [3]. Adolescents who experience suicidal ideation and planning are at increased risk of attempted suicide; previous history of attempted suicide represents the single strongest risk for death by suicide [4–7].

The growing body of evidence suggests that suicide is among the top 12 causes of deaths among persons aged 10–24 years within sub-Saharan Africa [8, 9]. Evidence of recent regional systematic reviews and meta-analyses indicates that compared to other low- and middle-income regions of the world, the 12-month prevalence estimates of suicidal ideation (20.4, 95% confidence interval [CI] = 17.3, 23.6), and suicidal planning (23.7, 95%CI = 19.1, 28.3) are higher among school-going adolescents in sub-Saharan Africa [10, 11], while the pooled 12-month prevalence estimates of suicidal attempt among school-going adolescents in sub-Saharan Africa (19.3, 95%CI = 14.2, 24.4) is comparable to what is reported in other LAMICs within the Western Pacific region (20.5, 95%CI = 14.3, 26.7) [11]. The most recent systematic review of primary studies involving school-going, household and ‘out-of-school’ adolescents in sub-Saharan Africa has reported a comparable pooled 12-month prevalence estimates of suicidal attempt (median = 17.1%, interquartile range [IQR] = 11.5–25.6%) [12]. In Western sub-Saharan Africa, where Liberia is located, the pooled 12-month prevalence estimates of suicidal attempt is relatively higher (Median = 24.3%, IQR = 16.9–27.9) [12]. Comparatively, whereas higher estimates of suicidal ideation, planning and attempt have been generally reported among female adolescents [13], no clear gender and age differences have been reported from sub-Saharan Africa [10, 12, 14].

Consistent with evidence form high-income countries positing suicidal behaviour as a complex phenomenon [1, 5, 6, 15], available studies from sub-Saharan Africa suggest that the factors associated with suicidal ideation, planning and attempt among school-going adolescents are multiple, but also multi-contextual, existing at the individual level (e.g., female gender, alcohol and substance use, anxiety, depression and psychiatric problems), in the family (e.g., conflict with parents, food insecurity, child marriage), school (e.g., bullying victimisation, poor academic performance, truancy), peer and social relationships (e.g., sexual abuse victimisation, physical attack/abuse victimisation), and within the larger community context (e.g., exposure to violence and war trauma, poverty) [10, 12, 14].

Considering the complex and multi-layered nature of the factors associated with suicidal behaviours, leading researchers and the WHO have recommended the application of the socio-ecological framework to the study and understanding of suicidal behaviours among young people [1, 16–18]. The socio-ecological framework suggests that the associated factors of suicidal behaviour are as a result of the interplay among multiple factors at multiple sectors and layers of an individual’s distal and proximal environment. Thus, the risks and protective factors of suicidal behaviours vary beyond the individual adolescent’s personal lifestyle and health histories, family and school circumstances, to include social and broader cultural factors.

### Aims of the study

It is noteworthy that both extant and recent global and regional systematic reviews of studies reporting suicidal behaviours among adolescents have not found any published primary studies from Liberia [10–14, 19], although local media reports show disturbing trends of adolescent suicide in the country [20, 21]. Therefore, in the current study we draw on data from the 2017 Liberia Global School-based Student Health Survey (GSHS) conducted nationwide among secondary school students to:

I Estimate the 12-month prevalence of suicidal behaviours (ideation, planning, and attempt) among school-going adolescents in Liberia.
J Identify the commonly reported psychosocial factors associated with suicidal behaviours (ideation, planning, and attempt) among school-going adolescents in Liberia.

## Methods

### Context and data source

The design and development of this study draw on data from the 2017 Liberia GSHS conducted by the WHO and the Centres for Disease Prevention (CDC) of the United States [22]. Liberia is an Anglophone West African country categorised as a low-income country, with a low human development index (HDI rank of 176) [23, 24]. Liberia’s general population is about 4.8 million, and the composition is relatively young, as 63% of the population is aged less than 25 years, and 32.8% is 10–24 years old [25, 26]; the mean years of schooling is 4.7 years [23]. Although the penal law of Liberia criminalises attempted suicide [27], suicide remains a leading cause of death within the country’s general population [28].

### Study design and sample

The GSHS is a cross-sectional national survey conducted in interested WHO member countries to evaluate behavioral health factors among youth in school. Data were collected by means of a self-administered questionnaire. More information on the methodology and the topics addressed by the survey are available on the WHO website [22].

Briefly, each country’s GSHS questionnaire consisted of validated items assessing the behavioural risks and protective factors across multiple areas of functioning (e.g., personal lifestyle, family relationships, and school environment) among school-going adolescents. The 2017 Liberia GSHS questionnaire is freely available on the WHO’s website: https://www.who.int/ncds/surveillance/gshs/Liberia_2017_GSHS_Questionnaire.pdf). After obtaining ethical approval and permissions from the relevant authorities and parents/guardians, data for the GSHS are collected from a nationally representative sample of secondary school students. Students who voluntarily consent to complete the survey record their own answers on a computer scannable form distributed by trained staff during one standard class period. The survey is anonymous; individually identifiable information or school-level identifiers are not collected. About 2 years after the data are gathered, clean data files are made freely available to the public [22]. We obtained the data analysed in this study freely from the WHO website: https://www.who.int/ncds/surveillance/gshs/liberia/en/. In analysing and reporting this cross-sectional data, we have been guided by the recommendations of Strengthening the Reporting of Observational Studies in Epidemiology (STROBE) [29].

### Sampling

In all countries participating in the GSHS, a two-stage approach is used to generate a nationally representative sample of in-school adolescents who are in the target age ranges. The first stage consists of a cluster sampling design in which schools are randomly sampled from a list of all schools in the country using a probability proportionate to size (PPS) method. This method ensures that the participants represent the geographic diversity of the country. In the second stage of the sampling process in each country, several classrooms that include high proportions of students from the targeted age groups are sampled for inclusion from within each of the participating schools. All students in each selected school had an equal chance of being selected for the study. Participants for the current study were grades 7–12 students sampled from selected schools in Liberia. Due to the complex sampling design, numerical weights were applied to each student record to enable generalization of study results to the student population of Liberia. Of the sampled schools, 98% participated in the study and the student response rate was 73%, whilst the overall response rate was 71%.

### Measures

#### Outcome variables

Considering that the focus of our study was on suicidal behaviours, we selected three main outcome measures from the data: suicidal ideation, suicidal planning, and suicidal attempt. Each of these three outcome variables was a single-item measure. Specifically, the item, “during the past 12 months, did you ever seriously consider attempting suicide?” was used to assess suicidal ideation, while suicidal planning was measured with the question, “during the past 12 months, did you make a plan about how you would attempt suicide?”. The responses were dichotomised as “yes” (1) or “no” (0). Suicidal attempt was measured with the question “during the past 12 months, how many times did you actually attempt suicide?” The responses for this question were “0”, “1”, “2 or 3”, “4 or 5”, and “6 or more times”. Consistent with most secondary analyses of the GSHS data from sub-Saharan Africa, we applied a binary recoding to the suicidal attempt variable, no attempt (0) and one or more attempts (1) for analysis [12].

#### Exposure variables

In order to facilitate comparison, the selection of exposure variables was informed by previous studies of correlates, risks, and protective factors for suicide behaviours in adolescents within sub-Saharan African countries [12]. Demographic variables included were age, school grade, and gender. Other variables including school, family, as well as personal and lifestyle factors were used to determine their predictive effects on the three outcome variables (suicide ideation, suicide attempts and suicide plans). The specific variables and their survey questions used, and coding used for the statistical analysis are presented as Supplementary Material (e-Table 1).

#### Statistical analyses

Analyses were performed with Stata 14.0 statistical software (StataCorp LP, College Station, Texas, USA). Sample weights were used in all analyses, so results are generalisable to the population. Univariate analysis involving frequencies and proportions were used to estimate the 12-month prevalence and 95% confidence intervals of suicidal ideation, planning, and attempt. In the primary analyses, chi-square tests were performed to examine the bivariate relationships between the exposure and outcomes variables. Exposure variables that demonstrated significant differences between those who reported suicide behaviours and those who did not at or below the 0.05 level (*p* < 0.05) threshold were then entered into logistic regression models in the second step.

The second step involved creating chunk-wise logistic regression models separately for risk factors and for protective factors for suicide behaviours. Three logistic regression models were developed for each outcome variable (suicidal ideation, planning, and attempt). The risk factor models included age, sex, school grade and risk factors significant in the bivariate analysis. The protective factor models included age, sex, and significant protective factors in the bivariate analysis. All variables from these two logistic regression models (both risk and protective factors) were then entered simultaneously into final logistic regression models to determine factors associated with suicide behaviours among the survey participants. Demographic variables (age, sex and school grade) were included in all logistic regression models.

## Results

### Participant characteristics

A total of 2744 students participated in the study, including 1382 (50.3%) male, 1253 (45.7%) female students and 109 (4%) participants with missing responses for gender. More than half (57.3%) of the participants reported to having been physically attacked on one or more occasions in the past 12 months. Additionally, 87.4% of them had many close friends, 36.8% felt supported by their peers and 20% spent 3 or more hours a day engaged in leisure or sedentary behaviours.

### Prevalence estimates

As shown in Table 1 the 12-month prevalence estimates of suicidal ideation, planning, and attempt were comparable between females and males. Overall, whereas approximately, 2 out of 10 adolescents reported suicidal ideation during the previous 12 months, 3 in 10 adolescents reported each of suicidal planning and attempt during the period. Of the adolescents who reported suicidal attempt, 16.5% reported a single episode and 17.2% repeated the attempts at suicide during the previous 12 months.

**Table 1:** Twelve month prevalence estimates of suicidal behaviour (ideation, planning, and attempt)

	Overall	Male	Female
	% (95% CI)	% (95% CI)	% (95% CI)
Suicidal ideation	26.8 (25.1–28.5)	25.5 (23.2–27.9)	27.0 (24.5–29.6)
Suicidal planning	36.5 (34.6–38.4)	36.7 (34.1–39.4)	35.4 (32.7–38.2)
Suicidal attempt	33.7 (31.9–35.6)	33.8 (31.2–36.4)	33.4 (30.7–36.2)

### Bivariate associations

Bivariate findings are presented in Table 2. Generally, most of the variables showed significant bivariate associations with suicidal behaviours. However, among the demographic variables, age was strongly associated with suicidal behaviours; adolescents aged 18 and above were more likely than those ages 17 and younger to report suicidal behaviours during the previous 12 months. Similarly, among the personal and lifestyle variables, alcohol drunkenness showed the strongest relationship with suicidal attempt (χ^2^_(1)_ = 49.66, *p* < 0.001), while cannabis use was strongly related to suicidal ideation (χ^2^_(1)_ = 35.42, *p* < 0.001), planning (χ^2^_(1)_ = 18.79, *p* < 0.001), and attempt (χ^2^_(1)_ = 116.7, *p* < 0.001). Among the school-related factors, adolescent victims of bullying and physical attack were more likely to report all three domains of suicidal behaviour, whereas those who were truant were more likely to report suicidal attempt (χ^2^_(1)_ = 25.37, *p* < 0.001). Among the family-related factors, although food insecurity was related to suicidal ideation, adolescents who reported food insecurity were more likely to report suicidal planning (χ^2^_(1)_ = 27.88, *p* < 0.001) and attempt (χ^2^_(1)_ = 23.18, *p* < 0.001). Parental monitoring and parental understanding were significantly associated with suicide plans and attempts but not suicidal ideation. None of the suicidal behaviours differed according to level of parental supervision, and parent intrusion of privacy was only associated with suicide attempt.

**Table 2:** Bivariate analysis of the factors associated with suicidal behaviours

Variable	Suicidal Ideation (***n*** = 682)				Suicidal planning (***n*** = 928)				Suicidal attempt (***n*** = 861)			
	No	Yes	χ^2^	*p*-value	No	Yes	χ^2^	*p*-value	No	Yes	χ^2^	*p*-value
	n (%)	n (%)			n (%)	n (%)			n (%)	n (%)		
**Demographics**												
Gender			0.72	.397			0.46	.499			0.04	.843
Male	973 (74.5)	333 (25.5)			821 (63.3)	476 (36.7)			861 (66.2)	439 (33.8)		
Female	849 (73.0)	314 (27.0)			745 (64.6)	408 (35.4)			772 (66.6)	387 (33.4)		
Age (in years)			21.24	<.001			11.39	.001			19.85	<.001
≤ 17 years	957 (77.7)	275 (22.3)			833 (67.1)	408 (32.9)			874 (70.5)	366 (29.5)		
≥ 18 years	869 (69.5)	381 (30.5)			746 (60.6)	485 (39.4)			771 (62.0)	472 (38.0)		
School grade			0.18	.673			0.53	.468			0.80	.371
Grade 7 to 9	1037 (73.6)	371 (26.4)			898 (64.1)	503 (35.9)			937 (67.0)	461 (33.0)		
Grade 10 to 12	807 (72.9)	300 (27.1)			694 (62.7)	413 (37.3)			731 (65.3)	388 (34.7)		
**Personal factors**												
Alcohol drunkenness			15.41	<.001			4.77	.029			49.66	<.001
Yes	310 (67.7)	148 (32.3)			269 (60.5)	176 (39.5)			249 (53.5)	216 (46.5)		
No	1422 (76.6)	435 (23.4)			1228 (66.0)	634 (34.0)			1308 (70.7)	542 (29.3)		
Leisure-time sedentary behaviour			3.00	.083			4.85	.028			14.10	<.001
≥ 3 h/day	324 (71.5)	129 (28.5)			273 (60.5)	178 (39.5)			280 (59.8)	188 (40.2)		
< 3 h/day	1397 (75.5)	454 (24.5)			1220 (66.1)	627 (33.9)			1273 (69.0)	573 (31.0)		
Cannabis use			35.42	<.001			18.79	<.001			116.7	<.001
Yes	96 (56.5)	74 (43.5)			81 (50.0)	81 (50.0)			58 (32.6)	120 (67.4)		
No	1600 (76.9)	480 (23.1)			1396 (66.8)	694 (32.2)			1489 (71.7)	587 (28.3)		
**School factors**												
Physical attack			15.85	<.001			23.64	<.001			69.07	<.001
Yes	994 (70.4)	418 (29.6)			842 (59.7)	568 (40.3)			848 (59.6)	575 (40.4)		
No	834 (77.5)	242 (22.5)			741 (69.2)	330 (30.8)			809 (75.5)	263 (24.5)		
Truancy			2.05	.152			1.46	.227			25.37	<.001
Yes	764 (73.0)	282 (27.0)			669 (63.6)	383 (36.4)			661 (62.2)	401 (37.8)		
No	954 (75.7)	307 (24.3)			829 (66.0)	427 (34.0)			908 (72.1)	352 (27.1)		
Peer support			0.97	.324			0.01	.948			0.01	.966
Yes	633 (75.7)	203 (24.3)			542 (64.8)	295 (35.2)			565 (67.2)	276 (32.8)		
No	1056 (73.8)	374 (26.2)			926 (64.9)	501 (35.1)			968 (67.3)	471 (32.7)		
Close friends			13.76	<.001			9.19	.002			0.80	.370
Yes	1627 (74.8)	547 (25.2)			1412 (65.2)	755 (34.8)			1455 (67.0)	718 (33.0)		
No	195 (64.8)	106 (35.2)			171 (56.3)	133 (43.7)			197 (64.4)	109 (35.6)		
Bullying victimisation			52.95	<.001			33.32	<.001			160.5	<.001
Yes	718 (66.3)	365 (33.7)			620 (58.2)	445 (41.8)			583 (53.3)	511 (46.7)		
No	897 (80.0)	224 (20.0)			794 (70.0)	340 (30.0)			888 (78.7)	240 (21.3)		
**Family factors**												
Parental supervision			0.01	.968			0.06	.801			0.03	.854
Yes	782 (74.5)	268 (25.5)			675 (64.4)	374 (35.6)			708 (67.1)	348 (32.9)		
No	933 (74.4)	321 (25.6)			810 (64.9)	439 (35.1)			850 (67.4)	411 (32.6)		
Parental understanding			0.25	.616			7.02	.008			4.63	.031
Yes	709 (75.3)	233 (24.7)			637 (67.9)	301 (32.1)			654 (69.7)	285 (30.3)		
No	979 (74.3)	338 (25.7)			820 (62.5)	492 (37.5)			869 (65.3)	461 (34.7)		
Parental monitoring			0.39	.535			9.38	.002			4.12	.042
Yes	649 (75.6)	209 (24.4)			590 (69.3)	261 (30.7)			601 (70.4)	253 (29.6)		
No	1018 (74.5)	349 (25.5)			855 (63.0)	503 (37.0)			910 (66.2)	464 (33.8)		
Food insecurity			8.55	.003			27.88	<.001			23.18	<.001
Yes	271 (67.6)	130 (32.4)			211 (52.2)	193 (47.8)			225 (56.3)	175 (43.7)		
No	1544 (74.6)	525 (25.4)			1361 (66.0)	700 (34.0)			1421 (68.7)	649 (31.3)		
Parent intrusion of privacy			0.31	.577			2.43	.119			8.77	.003
Yes	331 (73.4)	120 (26.6)			279 (61.6)	174 (38.4)			283 (61.3)	179 (38.7)		
No	1368 (74.7)	464 (25.3)			1196 (65.5)	630 (34.5)			1257 (68.5)	578 (31.5)		

Note: χ^2^ = chi square

### Multivariate associations

Results of the logistic regression models are presented in Table 3, stratified by (assumed) risk and protective factors of suicidal ideation, planning, and attempt.

**Table 3:** Multivariate associations

Variables in models	Suicidal ideation				Suicidal planning				Suicidal attempt			
	*β*	aOR	95% CI	*p*-value	*β*	aOR	95% CI	*p*-value	*β*	aOR	95% CI	*p*-value
**Logistic regression for risk factors**												
*Demographic variables*												
Gender												
Female (reference)		1.00				1.00				1.00		
Male	0.068	1.07	0.83, 1.38	0.596	0.176	1.19	0.95,1.49	0.123	0.100	1.11	0.87, 1.40	0.407
Age (in years)												
≤ 17 (reference)		1.00				1.00				1.00		
≥ 18	**0.299**	**1.35**	**1.02, 1.79**	**0.039**	0.123	1.13	0.88,1.46	0.346	0.183	1.20	0.92, 1.57	0.184
School grade												
Grade 7–9 (reference)		1.00				1.00				1.00		
Grade 10–12	−0.154	0.86	0.65, 1.14	0.285	−0.119	0.89	0.69, 1.14	0.356	−0.018	0.98	0.75, 1.28	0.893
*Personal and lifestyle factors*												
Alcohol drunkenness	0.063	1.06	0.76, 1.49	0.716	0.047	1.05	0.77, 1.42	0.764	0.307	1.36	1.00, 1.85	0.051
Leisure-time sedentary behaviour	0.227	1.26	0.92, 1.71	0.149	0.230	1.26	0.95, 1.66	0.103	**0.304**	**1.36**	**1.02, 1.81**	**0.038**
Cannabis use	0.431	1.54	0.94, 2.51	0.085	0.277	1.32	0.81, 2.14	0.263	**1.010**	**2.75**	**1.69, 4.47**	**< 0.001**
*School environmental factors*												
Truancy	−0.067	0.93	0.72, 1.21	0.607	0.041	1.04	0.83, 1.31	0.726	0.179	1.20	0.94, 1.52	0.143
Bullying victimisation	**0.635**	**1.89**	**1.45, 2.45**	**< 0.001**	0.359	**1.43**	**1.14, 1.80**	**0.002**	**0.797**	**2.22**	**1.74, 2.83**	**< 0.001**
Physical attack	0.217	1.24	0.96, 1.61	0.102	0.234	**1.26**	**1.01, 1.59**	**0.046**	**0.553**	**1.74**	**1.36, 2.22**	**< 0.001**
*Family-related factors*												
Parental intrusion of privacy	0.029	1.03	0.75, 1.40	0.855	0.109	1.12	0.85, 1.47	0.437	0.224	1.25	0.94, 1.67	0.127
Food insecurity	**0.394**	**1.48)**	**1.06, 2.08**	**0.022**	**0.523**	**1.69**	**1.24, 2.30**	**0.001**	**0.475**	**1.61**	**1.16, 2.22**	**0.004**
**Logistic regression for protective factors**												
Demographic variables												
Gender												
Female (reference)		1.00				1.00				1.00		
Male	0.041	1.04	0.85, 1.29	0.699	0.088	1.09	0.90, 1.32	0.363	0.027	1.03	0.85, 1.25	0.783
Age (in years)												
≤ 17 (reference)		1.00				1.00				1.00		
≥ 18	**0.449**	**1.56**	**1.24, 1.98**	**< 0.001**	**0.295**	**1.34**	**1.08, 1.66**	**0.007**	**0.394**	**1.48**	**1.19, 1.84**	**< 0.001**
School grade												
Grade 7–9 (reference)		1.00				1.00				1.00		
Grade 10–12	−0.164	0.85	(0.67, 1.07	0.173	0.002	1.00	0.81, 1.24	0.987	−0.026	0.97	0.78, 1.21	0.814
*School environment factors*												
Peer support	−0.065	0.94	0.75, 1.17	0.572	0.079	1.08	(0.88, 1.33)	0.444	0.030	1.03	0.84, 1.27	0.773
Close friends	**−0.488**	**0.61**	**0.46, 0.83**	**0.001**	**−0.367**	**0.69**	**0.52, 0.92**	**0.011**	−0.107	0.90	0.67, 1.20	0.470
*Family factors*												
Parent supervision	0.203	1.23	0.97, 1.55	0.091	**0.248**	**1.28**	**1.04, 1.59**	**0.023**	**0.278**	**1.32**	**1.06, 1.64**	**0.012**
Parental understanding	−0.044	0.96	0.75, 1.21	0.718	**−0.254**	**0.78**	**0.62, 0.96**	**0.021**	**−0.238**	**0.79**	**0.63, 0.98**	**0.034**
Parental monitoring	−0.079	0.92	0.73, 1.17	0.510	**−0.254**	**0.78**	**0.63, 0.96**	**0.021**	−0.214	0.81	0.65, 1.01	0.056
**Final logistic regression model for all risk and protective factors**												
Demographic variables												
Gender												
Female (reference)		1.00				1.00				1		
Male	0.148	1.16	0.89, 1.51	0.273	0.169	1.18	0.94, 1.50	0.158	0.123	1.13	0.88, 1.45	0.334
Age (in years)												
≤ 17 (reference)		1.00					1.00			1.00		
≥ 18	0.235	1.26	0.94, 1.71	0.123	0.077	1.08	0.83, 1.41	0.572	0.201	1.22	0.92, 1.63	0.167
School grade												
Grade 7–9 (reference)		1.00					1.00			1.00		
Grade 10–12	−0.095	0.91	0.68, 1.22	0.531	−0.029	0.97	0.74, 1.27	0.830	−0.002	1.00	0.75, 1.32	0.987
*Risk factors*												
Alcohol drunkenness	0.148	1.16	0.81, 1.65	0.412	0.181	1.20	0.87, 1.65	0.270	**0.371**	**1.45**	**1.04, 2.01**	**0.027**
Leisure-time sedentary behaviour	0.288	1.33	0.97, 1.84	0.080	**0.292**	**1.34**	**1.01, 1.79**	**0.048**	**0.391**	**1.48**	**1.09, 2.00**	**0.011**
Cannabis use	0.325	1.38	0.82, 2.33	0.223	0.228	1.26	0.75, 2.10	0.387	**1.015**	**2.76**	**1.62, 4.69**	**< 0.001**
Truancy	−0.020	0.98	0.75, 1.29	0.883	0.083	1.09	0.85, 1.38	0.497	0.241	1.27	0.99, 1.64	0.063
Bullying victimisation	**0.646**	**1.91**	**1.45, 2.51**	**< 0.001**	**0.402**	**1.49**	**1.18, 1.90**	**0.001**	**0.825**	**2.28**	**1.77, 2.94**	**< 0.001**
Physical attack	0.289	1.31	0.99, 1.72	0.056	0.199	1.22	0.96, 1.55	0.108	**0.591**	**1.81**	**1.39, 2.34**	**< 0.001**
Parental intrusion of privacy	0.109	1.12	0.79, 1.57	0.529	0.240	1.27	0.94, 1.72	0.120	0.233	1.26	0.91, 1.74	0.159
Food insecurity	**0.435**	**1.55**	**1.09, 2.20**	**0.015**	**0.600**	**1.82**	**1.32, 2.51**	**< 0.001**	**0.528**	**1.70**	**1.20, 2.39**	**0.002**
*Protective factors*												
Peer support	−0.077	0.93	0.70, 1.23	0.590	0.039	1.04	0.81, 1.33	0.760	0.155	1.17	0.90, 1.52	0.251
Close friends	−0.309	0.73	0.49, 1.10	0.138	**−0.556**	**0.57**	**0.40, 0.82**	**0.002**	**−0.475**	**0.62**	**0.42, 0.92**	**0.016**
Parent supervision	0.194	1.21	0.90, 1.64	0.204	0.266	1.25	0.96, 1.64	0.097	**0.393**	**1.48**	**1.11, 1.97**	**0.007**
Parental understanding	0.069	1.07	0.80, 1.44	0.650	−0.154	0.86	0.66, 1.12	0.255	**−0.294**	**0.75**	**0.56, 0.99**	**0.043**
Parental monitoring	−0.115	0.89	0.66, 1.20	0.455	**−0.273**	**0.76**	**0.58, 0.99**	**0.048**	−0.203	0.82	0.61, 1.09	0.165

Note: *β* beta value, *aOR* adjusted odds ratio, *CI* Confidence Interval; statistically significant results are in bold face

#### Risk factors for suicidal behaviour

In the final adjusted logistic models, no variable showed unique associations with the increased odds of suicidal ideation. However, bullying victimisation and food insecurity emerged as significant exposure factors associated with increased odds of suicidal ideation, planning, and attempt. Leisure-time sedentary behaviour was associated with increased odds of suicidal planning (OR: 1.34, 95% CI: 1.01–1.79; *p* = 0.048) and attempt (OR: 1.48, 95% CI: 1.09–2.00; *p* = 0.011), while cannabis use (OR: 2.76, 95% CI: 1.62–4.69; *p* < 0.001), being physically attacked (OR: 1.81, 95% CI: 1.39–2.34; p < 0.001), parental supervision (OR: 1.48, 95% CI: 1.11–1.97; *p* = 0.007), and alcohol drunkenness (OR: 1.45, 95% CI: 1.04–2.01; *p* = 0.027) were associated with increased odds of suicidal attempt only. Interestingly, having many close friends (OR: 0.57, 95% CI: 0.40–0.82; *p* = 0.002), and parental monitoring (OR: 0.76, 95% CI: 0.58–0.99; *p* = 0.048) were uniquely associated with the increased odds of suicidal planning.

#### Protective factors of suicidal behaviour

Similarly, no variable showed unique associations with reduced odds of suicidal ideation only in the final adjusted logistic models. However, parental understanding (OR: 0.75, 95% CI: 0.56–0.99; *p* = 0.043) and having a smaller number of close friends (OR: 0.62, 95% CI: 0.42–0.92; *p* = 0.016) were uniquely associated with reduced odds of suicidal attempt only.

Notably, there was no gender associations with suicidal behaviours – a plausible indication that the factors presenting as precipitants of suicidal behaviour among school-going adolescents in Liberia may be commonly distressing for both boys and girls.

## Discussion

This study has shown two key findings: firstly, approximately, 2 out of 10 school-going adolescents in Liberia report suicidal ideation during the previous 12 months, while 3 in 10 adolescents report each of suicidal planning and attempt during the same period; secondly, personal risky health behaviours and social adversities are associated with increased odds of suicidal behaviours, whereas supportive social and family-related factors are associated with reduced odds of suicidal behaviours among school-going adolescents in Liberia.

### Prevalence of suicidal behaviours

Compared to the range of estimates of suicidal ideation, planning, and attempt from Western sub-Saharan Africa and countries within sub-Saharan Africa generally [10–12, 14, 19, 30], the estimates of suicidal behaviours in the present study are higher. Still, the school-going adolescents in the present study reported higher estimates of suicidal behaviours than estimates of suicidal ideation (11, 95%CI = 9–14) and attempt (6, 95%CI = 4–8) among the general household-based adult population in Liberia [31]. In one breadth, the evidence of higher estimates of suicidal behaviours in this study may not be entirely surprising, considering Liberia’s recent history of conflict and exposure to traumatic events including violence and the attendant effects of wars, like sexual violence and displacement – which have been found to be strong risk factors for suicidal behaviours among both young people and adults [28, 31, 32]. In another breadth, the use of single-item measure to assess each of suicidal ideation, planning, and attempt warrants a cautious interpretation of the reported estimates, as single-item measures of suicidal behaviours often result in higher estimates and misclassification of suicidal behaviours [33]. However, beyond these two cautious interpretations, the evidence of higher estimates of suicidal behaviours and the pioneer nature of the present study clearly point to the need for further evidence through expansive epidemiological research to clarify the extent of suicidal behaviours among school-going adolescents in Liberia.

Lastly, it is also not clear why the estimate of suicidal attempt is higher than suicidal ideation in the current study – an observation that is counterintuitive, unconventional and inconsistent with the suicide pathway or process model [1, 5, 34]. Although similar results have been reported by previous GSHS from other sub-Saharan African countries, including Benin [35], Ghana [36], and Malawi [37], we suspect that this counterintuitive evidence may be more attributable to the measurement of suicidal behaviours – in particular, the use of single-item measures in the GSHS. Also, beyond the evidence of the present study, we suspect that the higher estimate of suicidal attempt (than suicidal ideation) may be due to impulsivity. Available evidence from high-income countries suggests that among young people, impulsivity can ‘facilitate’ the onset of suicidal attempt, without prior suicidal ideation [34, 38, 39].

### Factors associated with suicidal behaviours

This study has found that increased odds of suicidal behaviours is associated with personal risky health behaviours: leisure-time sedentary behaviour, cannabis use, and alcohol drunkenness. For example, this study shows that adolescents who used cannabis were about three times more likely to report suicidal attempt than those who did not report cannabis use. Consistent with evidence from high-income countries [40, 41], these risky health behaviours and their strong associations with increased odds of suicidal behaviours have been found in nearly all the GSHS across the African region [12, 42, 43]. The evidence supports the consistent observation that young people in Africa still face multiple challenges that expose them to risky health behaviours [44–46]. For example, whereas underage use of cannabis and alcohol is illegal in African countries, the enforcement of such laws remains a critical challenge, with retailers selling out alcohol to minors [44, 47, 48]. A recent review of the literature has reported that in Anglophone West Africa, alcohol, cannabis and other unconventional substances are commonly misused by young people in post-conflict countries, including Liberia [49]. In Liberia, peer influence has also been implicated for alcohol and substance use among school-going adolescents [50]. Alcohol and substance use has been observed to have the combined effects of complicating the course of depression and impairing judgement of a person experiencing depression, and at the same time resulting in high impulsivity which leads to acute life-threatening behaviours such as attempted suicide [51]. Leisure-time sedentary behaviour is now a common problem across most low- and middle-income countries, including those in Africa [52]. As a risk factor, leisure-time sedentary behaviour is associated with elevated levels of depressive symptoms, which in turn increases that risk for suicidal behaviour among young people [43, 53].

This study’s finding that the social adversities of bullying victimisation, being physically attacked, and food insecurity are associated with increased odds of suicidal behaviours is consistent with evidence from the GSHS in other African countries, including Benin, Ghana, and Mauritania in West Africa [12, 35, 36, 54, 55]. Evidence suggests that social and interpersonal adversities could result in internalising problems such as self-blame and self-dislike, depression, and feelings of shame and guilt – that may engender self-harm and suicidal tendencies in young people [14]. As observed in other African countries, adolescents who go hungry may experience heightened distraction, irritability and increased emotional responsiveness, which could result in suicidal tendencies [36, 55].

The finding that parental monitoring, parental supervision, and having many close friends are associated with increased odds of suicidal planning is counterintuitive and surprising. The finding is inconsistent with existing Western life-course models that suggests that larger social networks, parental monitoring and supervision are strong predictors of positive health and behavioural outcomes among adolescents across cultures [44, 56]. Although similar evidence has been reported among in-school adolescents in Ghana [36, 57], perhaps, the Liberian situation is different; thus, further context-relevant evidence is needed to clarify the nature of these associations among school-going adolescents in Liberia.

An encouraging finding of the present study is that the reduced odds of suicidal attempt are associated with supportive social and family-related factors: parental understanding and having a smaller number of close friends. This supports evidence from Ghana [36], Namibia [58], South Africa [59], Tanzania [60] and countries in the Caribbean [61], that parental support and parental understanding are associated with reduced chances of suicidal behaviours among adolescents. Evidence suggests that parental understanding tends to be associated with involvement in healthy behaviours and reduced engagement in high risk, life-threatening behaviours, including suicide among adolescents [62, 63]. Evidence from Seychelles [64] suggests that having a smaller number of close friends is associated with higher odds of suicidal ideation; interestingly, findings from Ghana [36], and Malawi [37] also suggests that having more close friends is associated with higher odds of suicidal attempt among school-going adolescents. However, our study shows that having a smaller number of close friends is associated with reduced odds of attempted suicide. Perhaps, future qualitative studies using robust designs may prove useful in clarifying this association among school-going adolescents in Liberia.

### Implications and recommendations

Consistent with the socio-ecological framework of suicidal behaviours among young people, the associated factors of suicidal behaviours found in the present study are multi-layered and multi-level in nature, ranging from personal/lifestyle factors, family relationships, school factors and broader interpersonal relationship factors. Thus, the key findings of this study have some implications for multi-sectorial and multi-contextual intervention and prevention efforts, and policy directions. On the intervention and prevention front, suicidologists and other suicide prevention professionals may collaborate with schools and parent-teacher associations for targeted prevention involving educating parents on supportive parenting and specific parenting behaviours that protect adolescents against self-destructive behaviours, plus spotting warning signs of suicidal behaviours and how to help adolescents experiencing suicidal tendencies. This suggestion for youth-targeted suicide prevention efforts is also important because although young people in post-conflict Libera are faced with many negative mental health outcomes, currently, infrastructure and other support systems against mental health problems among young people are unavailable in the country [65, 66].

Relatedly, as reported by a recent review, although bullying is pervasive in schools within LAMICs, anti-bullying policies are lacking, and where they exist, implementation remains a problem [67]. Drawing on the evidence of the present study, we strongly recommend school-based anti-bullying and anti-violence polices across secondary schools in Liberia, with effective mechanisms in place for implementation. Similarly, government must prioritise the enforcement of the laws regulating the sale and use of alcohol, cannabis and other drugs, to prevent access by minors and students in Liberia. Consistent with recent calls by key stakeholders, including the United Nations World Food Programme [68, 69], the evidence of the present study underscores the need for the government of Liberia to consider expanding the school feeding programme across all schools in the country, while considering sound, sustainable poverty reduction strategies to support families to overcome food insecurity within the domestic environment. Universal prevention strategies against suicidal behaviours may consider teaching all school-going adolescents effective interpersonal and social skills for avoiding violence, bullying perpetration and victimisation, and resisting alcohol and substance use. Teaching emotion regulation and help-seeking behaviour could be helpful in avoiding suicidal planning and attempt among students.

### Strengths and limitations of the study

This study represents the first to draw on a nationally representative sample to report suicidal ideation, planning, attempt, and associated factors among secondary school students in post-conflict Liberia. The nationally representative sample included in the study makes the findings generalisable across school-going adolescents in the country. Nonetheless, some limitations are noteworthy. Although this study has accounted for some of the major interpersonal and social adversities associated with suicidal behaviours, our study fails to include key psychiatric variables – e.g., anxiety, depression – which have been identified as major associated factors of suicidal behaviours among adolescents [4, 12]. As reported elsewhere that there is a high tendency of nondisclosure of suicidal behaviour in research by students [70], even though the prevalence estimates of suicidal behaviour in the present study are relatively higher, it is possible that the criminalised and highly stigmatised status of suicidal behaviour in Liberia might have motivated some of the participants to provide guarded and socially desirable answers, particularly, in response to the survey question related to attempted suicide. As pointed out earlier, the use of single-item measures might have led to higher estimates and misclassification of previous suicidal behaviours assessed in this study [33]. Future studies may consider the use of multi-item behavioural measures or checklists to provide more precise results. The cross-sectional design used does not permit causal inference of results; longitudinal studies may be more useful in examining the pattern of suicidal behaviours and help to clarify the risks and protective factors related to suicidal ideation, planning and attempt among this young population. As recommended by a recent review from sub-Saharan Africa [12], in keeping with the fact that suicidal behaviours can be culture-specific and are embedded within the context of culture [71], qualitative studies are also needed to explore the individualised meanings and shared culture experiences that underlie suicidal behaviours among adolescents in Liberia [72].

## Conclusions

The relatively higher prevalence estimates of suicide behaviours and the multi-contextual nature of the associated factors warrant the need for the design and implementation of universal and multi-level, collaborative targeted intervention efforts towards the prevention of the onset of ideation, planning, and attempt, and the possible transition to deaths by suicide among school-going adolescents in Liberia.

## Supplementary Information

**Additional file 1: Table S1.** Socio-demographic and exposure variable derivation from the Liberia GSHS survey data, 2017.

## Data Availability

The datasets used and/or analysed during the current study are freely available from the WHO website: https://www.who.int/ncds/surveillance/gshs/liberia/en/. The 2017 Liberia GSHS questionnaire is also available freely on the WHO’s website: https://www.who.int/ncds/surveillance/gshs/Liberia_2017_GSHS_Questionnaire.pdf.

## Abbreviations

CDC: Centres for Disease Prevention
GSHS: Global School-based Student Health Survey
LAMICs: Low- and middle-income countries
WHO: World Health Organization
