# Supplementary material for: Suicidal behaviours among adolescents in Liberia
Source: BMC Psychiatry. 2020 Dec 1;20:572. doi: 10.1186/s12888-020-02985-3 (PMC7706245; doi:10.1186/s12888-020-02985-3)
Supplement: Supplementary file 1 — Additional file 1: Table S1. Socio-demographic and exposure variable derivation from the Liberia GSHS survey data, 2017. [file 12888_2020_2985_MOESM1_ESM.docx]

**Supplementary Material**

**e-Table 1:** Socio-demographic and exposure variable derivation from the Liberia GSHS survey data, 2017

| Variable | Survey question | Original response options | Recoded | Liberia, N = 2744; % missing |
| --- | --- | --- | --- | --- |
| Gender | What is your sex? | 1 = male; 0 = female | N/A | N= 2635, Missing = 109 (4.0%) |
| Age | How old are you? | 11–17 years (coded categorically) | N/A | N= 2661, Missing = 83 (3.0%) |
| School Grade | In what grade are you? | 1 = Grade 7 to 6 = Grade 12 | N/A | N= 2700, Missing = 44 (1.6%) |
| School truancy | During the past 30 days, how many days did you miss classes or school without permission? | 1 = 0 days to 5 = 10 or more days | 1 = 0 and 2–5 = 1 | N= 2460, Missing = 284 (10.3%) |
| Bullying victimisation | During the past 30 days, how many days were you bullied? | 1 = 0 days to 7 = all 30 days | 1= 0 and 2–7 = 1 | N= 2353, Missing = 391 (14.2%) |
| Physically attacked | During the past 12 months, how many times were you physically attack? | 1 = 0 times to 8 = 12 or more times | 1 = 0 and 2–8 = 1 | N= 2661, Missing = 83 (3.0%) |
| Close friends | How many close friends do you have? | 1 = 0 friends to 4 = 3 or more close friend | 1 = 0 and 2–4 = 1 | N= 2627, Missing = 117 (4.3%) |
| Peer support | During the past 30 days, how often were most of the students in your class kind and helpful? | 1 = never to 5 = always | 1–3 = 0 and 4–5 = 1 | N= 2409, Missing = 335 (12.2%) |
| Leisure-time sedentary behaviour | How much time do you spend during a typical or usual day sitting and watching television, playing computer games, talking with friends, or doing other sitting activities such as ludo, checkup, tag, cards, marbor, or scramble?” | 1= less than 1 hour per day to 6 = more than 8 hours per day | 1–2 = 0 and 3–6 = 1 | N= 2452, Missing = 292 (10.6%) |
| Alcohol drunkenness | During your life, how many times did you drink so  much alcohol that you were really drunk? | 1 = 0 times to 4 = 10 or more times | 1 = 0 and 2–7 = 1 | N= 2484, Missing = 260 (9.5%) |
| Cannabis use | During the past 30 days, how many times have you used marijuana (also called weed)?” | 1 = 0 days to 5 = all 30 days | 1 = 0 and 2–5 = 1 | N= 2383, Missing = 361 (13.2%) |
| Parental supervision | During the past 30 days, how often did your parents or guardians check to see if your homework was done? | 1 = never to 5 = always | 1–3 = 0 and 4–5 = 1 | N= 2453, Missing = 291 (10.6%) |
| Parental Intrusion of privacy | During the past 30 days, how often did your parents or guardians go through your things without your approval? | 1 = never to 5 = always | 1–3 = 0 and 4–5 = 1 | N= 2428, Missing = 316 (11.5%) |
| Parent Understanding | During the past 30 days, how often did your parents or guardians understand your problems and worries? | 1 = never to 5 = always | 1–3 = 0 and 4–5 = 1 | N= 2402, Missing = 342 (12.5%) |
| Parental monitoring | During the past 30 days, how often did your parents or guardians really know what you were doing you’re your free time? | 1 = never to 5 = always | 1–3 = 0 and 4–5 = 1 | N= 2351, Missing = 393 (14.3%) |
| Food insecurity | During the past 30 days, how often did you go hungry because there was not enough food in your home? | 1 = never to 5 = always | 1–3 = 0 and 4–5 = 1 | N= 2641, Missing = 103 (3.8%) |
